# Quantitative electrophysiological assessments as predictive markers of lower limb motor recovery after spinal cord injury: a pilot study with an adaptive trial design

**DOI:** 10.1038/s41394-022-00491-0

**Published:** 2022-02-24

**Authors:** Yin Nan Huang, El-Mehdi Meftah, Charlotte H. Pion, Jean-Marc Mac-Thiong, Julien Cohen-Adad, Dorothy Barthélemy

**Affiliations:** 1 Centre for Interdisciplinary Research in Rehabilitation of Greater Montreal (CRIR), Institut Universitaire de Réadaptation en Déficience Physique de Montreal, CIUSSS du Centre-Sud-de-l’Île-de-Montréal, Montreal, QC Canada; 2 Department of Medicine, Université de Montréal, Montreal, QC Canada; 3 School of Rehabilitation, Université de Montréal, Montreal, QC Canada; 4 Hôpital du Sacré-cœur de Montréal, CIUSSS Nord de l’ile de Montréal, Montreal, QC Canada; 5 NeuroPoly Lab, Institute of Biomedical Engineering, Polytechnique Montreal, Montreal, QC Canada; 6 Functional Neuroimaging Unit, CRIUGM, Université de Montréal, Montreal, QC Canada; 7 Mila—Quebec AI Institute, Montreal, QC Canada

**Keywords:** Predictive markers, Motor control

## Abstract

**Study design:**

Observational, cohort study.

**Objectives:**

(1) Determine the feasibility and relevance of assessing corticospinal, sensory, and spinal pathways early after traumatic spinal cord injury (SCI) in a rehabilitation setting. (2) Validate whether electrophysiological and magnetic resonance imaging (MRI) measures taken early after SCI could identify preserved neural pathways, which could then guide therapy.

**Setting:**

Intensive functional rehabilitation hospital (IFR).

**Methods:**

Five individuals with traumatic SCI and eight controls were recruited. The lower extremity motor score (LEMS), electrical perceptual threshold (EPT) at the S2 dermatome, soleus (SOL) H-reflex, and motor evoked potentials (MEPs) in the tibialis anterior (TA) muscle were assessed during the stay in IFR and in the chronic stage (>6 months post-SCI). Control participants were only assessed once. Feasibility criteria included the absence of adverse events, adequate experimental session duration, and complete dataset gathering. The relationship between electrophysiological data collected in IFR and LEMS in the chronic phase was studied. The admission MRI was used to calculate the maximal spinal cord compression (MSCC).

**Results:**

No adverse events occurred, but a complete dataset could not be collected for all subjects due to set-up configuration limitations and time constraints. EPT measured at IFR correlated with LEMS in the chronic phases (*r* = −0.67), whereas SOL H/M ratio, H latency, MEPs and MSCC did not.

**Conclusions:**

Adjustments are necessary to implement electrophysiological assessments in an IFR setting. Combining MRI and electrophysiological measures may lead to better assessment of neuronal deficits early after SCI.

## Introduction

Individuals with traumatic spinal cord injury (SCI) experience various long-term disabilities that lead to an impaired quality of life [1–4]. Development of treatments, although a high priority, is challenging: the ASIA/ISCoS International Standards for Neurological Classification of Spinal Cord Injury (ISNCSCI) [5–7] is used to subgroup patients. However, considerable variability is still observed within each group and the deficits sustained, long-term prognosis, and response to rehabilitation vary widely. Hence, there is a clear need for objective markers to accurately assess the characteristics of spinal cord lesions and complement current clinical tools.

As neuronal pathways are topographically distributed within each spinal segment, the ability to identify those impaired by the lesion would highlight the potential functional losses [8, 9]. Techniques such as magnetic resonance imaging (MRI) can assess the integrity of the spinal cord tissue and enable visualisation of the lesioned area [10–13]. Furthermore, electrophysiology has enabled the reliable assessment of neuronal pathways in humans [14, 15]. Notably, in individuals with SCI (>1 year post lesion), foot drop and decreased gait speed were correlated with lesions involving the corticospinal tract, whereas decreased balance control was correlated, in part, with impairment to the vestibulospinal system [16]. These studies emphasise that lesions in a specific pathway can be related to a specific set of functional deficits. Obtaining this information early in the rehabilitation process could guide treatment and optimise functional recovery.

However, although neurophysiological techniques have been shown to be valid measures of spinal cord excitability, they are not used for clinical decision making. The precise information they provide on actual neurological damage could support clinical decision making, particularly by predicting prognosis and recovery trajectory. For example, this information could be used as inputs into predictive outcome models, but this has not been tested to date. Attempts at predicting and modelling recovery using neurophysiological measurements have also been made with other diagnoses, such as stroke, and may lead to promising results [17–19].

The present study aimed to use combined multimodal electrophysiological techniques to clearly define the impact of lesions in specific pathways on sensorimotor control after SCI. We hypothesised that neural pathway assessments performed soon after SCI would provide timely characterisation of the spinal cord lesion and thereby predict long-term sensorimotor recovery.

Our objective was to test the feasibility and relevance of a multimodal electrophysiological assessment protocol in early intensive functional rehabilitation (IFR) within the rehabilitation setting. To this end, we tested a combination of approaches: (1) sensory pathways from the lower limbs, using the electrical perceptual threshold (EPT) of the S2 dermatome, (2) spinal networks below the injury level using electrically induced soleus muscle (SOL) H-reflex, and (3) the corticospinal tract, using transcranial magnetic stimulation (TMS) over the tibialis anterior muscle (TA) to represent the motor cortex. To complement electrophysiological data, analysis of MRI, obtained on admission to the acute care facility, was also performed to document the initial lesion.

## Methods

### Participants and setting

Participants provided informed, written consent for the experimental procedures of this pilot study, which was based on an adaptive trial design approved by the Research Ethics Boards of the Research Centre of Hôpital du Sacré-Coeur de Montréal and the Centre for Interdisciplinary Research in Rehabilitation of Greater Montreal. This study was conducted in accordance with the central tenets of the Declaration of Helsinki.

#### Participants with traumatic SCI

Five participants with traumatic SCI were recruited by a research nurse at the SCI unit of the acute care facility of the Hôpital du Sacré-Coeur de Montréal. Data was collected from May 2015 to January 2017. Patients were approached if they were hemodynamically stable, 18 to 60 years old, able to provide consent, able to follow instructions in French or English, and had no major cognitive deficits. Patients were excluded if they had sustained a cranial fracture at the time of the accident and if they had contraindications to TMS [20]. The baseline demographic and clinical characteristics of the patients are summarised in Table 1. The five participants included two men and three women (average age: 43.6 ± 11 years; range 30 to 55 years). All had lesions at the cervical level. Two were classified as having AIS A, two as AIS B, and one as AIS D at admission, according to the ASIA/ISCoS ISNCSCI [7].

**Table 1:** Characteristics of spinal cord injured patients.

ID	Sex	Age	AIS	Level cervical	Height (cm)	Weight (kg)	Medication	Delay between trauma and IFR assessment (months, days)	Delay between trauma and last assessment (years, months, days)
001SCI	M	55	A	C4–C5	168	79	Lyrica (125 mg)	4 m, 8 d	5 m, 2 d
002SCI	M	55	D	C2	173	68	Lyrica (100 mg)	1 m, 2 d	6 m, 2 d
003SCI	F	30	B	C4–C5	175	63.5	Lyrica (125 mg), Clonazepam	1 m, 1 d	1 y, 5 m
004SCI	F	39	B	C4	170	60.5	Lyrica (50 mg)	3 m, 6 d	1 y, 4 m
005SCI	F	39	A	C4–C5	173	71.5	Neurontin (40 mg)	2 m, 18 d	1 y, 5 m

*SCI* spinal cord injury, *cm* centimetre, *kg* kilogram.

#### Control participants

Eight control participants (5 M, 3 F; average age 34.9 ± 14.1 years; range 20 to 60 years) were also recruited. The same inclusion and exclusion criteria used for the SCI participants were applied.

### Experimental protocol

Each participant with SCI was assessed at two time points: during the early phase of IFR and in the chronic phase following the injury (≥6 months after SCI), when the patient had returned to the community or was awaiting placement in a specialised home. IFR treatments consisted of best practice physical therapy administered at the rehabilitation institute and performed by experienced physical therapists. The research team did not provide any interventions that would alter the therapists’ normal practice. Control participants were tested only once. The lower extremity motor score (LEMS) [21] and electrophysiological tests were undertaken at every assessment.

### Outcomes

#### Feasibility of measures

Feasibility was assessed using the following criteria: occurrence of adverse events (safety), total experiment duration, and completeness of the dataset. As this study was conducted with an adaptive trial design, feasibility was based on whether solutions could be found for each problem that arose.

#### Clinical assessment

Physicians performed clinical assessments of patients on hospital admission. SCI participants were classified on the completeness and level of their lesion according to the ISNCSCI [21]. To assess motor abilities, the AIS LEMSs were used by experienced physical therapists at all assessments. The LEMS of SCI participants in the chronic stage was regarded as their functional motor outcome, and the relationship between the motor outcome and the electrophysiological data collected in early IFR were examined.

#### Electrophysical assessment

Electrical activity was recorded from the soleus (SOL) and TA muscles of both lower limbs. Details of the recording have been described elsewhere [20, 22].

##### SOL H-reflex

To assess the SOL H-reflex, the participants sat on their wheelchairs or in a semi-reclined position on their hospital bed, keeping their head straight and looking forward. Both legs were evaluated. We stimulated the tibial nerve in the popliteal fossa using a 1-ms single-pulse monopolar electrical stimulation (constant-current Digitimer DS7, Digitimer Ltd HK; details provided in [23]). An H-reflex and muscular (M) response recruitment curve was constructed for all participants, without any contraction of the SOL muscle. Stimulus intensity was progressively increased every 5 s, in 2-mA increments, until the maximum H-reflex (H_max_) was obtained. The intensity was then increased in 10-mA increments until the maximal M response (M_max_) was reached. From this curve, the H_max_/M_max_ ratio and the H_max_ latency were noted.

##### Electrical perceptual threshold

Somatosensory pathways were tested by evaluating the EPTs of the S2 dermatome on both legs [24, 25]. S2 was tested as it is the last easily accessible dermatome of the lower limb. Using the same set-up as the one described for the SOL H-reflex, 1-ms square pulses were applied at the centre of the popliteal fossae at a 0.2 Hz frequency. The stimulation intensity was progressively increased and decreased manually to identify the participant’s ascending and descending perceptual thresholds, based on their verbal feedback. Two trials were performed for each threshold (ascending and descending), with the intensity being changed in 0.5 mA increments in the first trial and 0.1 mA in the second trial. The average of the ascending and descending perceptual thresholds was reported as the EPT for each participant.

##### Transcranial magnetic stimulation

TMS of the TA representation over M1 was performed while the participants sat on their wheelchairs, keeping their head straight and looking forward. TMS was not tested when SCI participants were assessed at the bedside. For SCI participants, both legs were tested, and for the control, only the right (*n* = 2) or left (*n* = 5) leg was evaluated (randomised). Single-pulse TMS (Magstim 200, Magstim Company Ltd., UK) was applied using a figure of 8-batwing coil over the leg area of the primary motor cortex, while SCI participants performed or attempted to perform a maximal contraction. Control participants were asked to perform a 10% maximal voluntary contraction. The coil’s optimal position, hotspot, and determination of motor threshold (MT) are described elsewhere [26] Ten TMS pulses were then randomly applied at 1.1 MT over a period of 2 min.

##### Magnetic resonance imaging

Anatomical images (T1-, T2- and T2*-weighted), routinely acquired by the acute care facility, were analysed. Using published methodologies [27], quantification of the maximal spinal cord compression (MSCC) at the compression site was determined using the following formula:

$${{{\mathrm{MSCC}}}} \,=\, \left( {1 \,-\, {{{\mathrm{di}}}}/\left( {{{{\mathrm{dr}}}} \,+\, {{{\mathrm{dc}}}}} \right)/2} \right) \,\ast\, 100\%$$

where, dr: diameter measured one level above the compression site; dc: measured one level below the compression site; di: measured at the level of the compression site. Hence, a higher MSCC reflects a larger compression, with 100% suggesting complete transection.

### Analysis

Feasibility outcomes were analysed using count data, descriptive statistics, rates (e.g. rates of occurrence), and narrative descriptions. LEMS results were reported as a score. The electrophysiological data were analysed separately for each leg. The EPT was reported as the mean intensity at which electrical stimulation was detected. The excitability of the SOL H-reflex was reported as H_max_/M_max_ ratio and H_max_ latency, and the excitability of the corticospinal tract was reported as presence, amplitude, latency of the motor evoked potential (MEP), and presence, latency, duration, and area of the silent period. The values were reported as mean ± SD. The MSCCs obtained from the MRI were reported as a score. To determine whether electrophysiological data obtained at the IFR time point could reflect the potential for long-term motor recovery of each patient, correlation analyses (Pearson’s correlation) were performed. Due to the low number of participants, summary and descriptive statistics tests were performed, and the effect size (Cohen’s *d*) was calculated, but the p value was not determined. Analyses were performed in SPSS.

## Results

### Feasibility

#### Safety

No adverse effects were observed. One SCI participant reported a light headache immediately after TMS application, but the headache was short lasting and not present on the following day. Two SCI participants (#1 and #5) were not tested with TMS, as one was mechanically ventilated and the other was apprehensive of the test.

#### Experiment duration

Data acquisition was performed in <3 h. This included preparation and resting periods. As a 3 h session could be tiresome at the early IFR time point, we performed data collection over two sessions for participants #3 and #4, which was well tolerated. The second session occurred once rehabilitation was ongoing (mid-IFR).

#### Data completeness

It was possible to conduct an assessment at the two time points in all participants. A complete dataset was collected for clinical data. Data collection for electrophysiological data was complete in the chronic stage but could not be completed for some participants in early IFR. Notably, for participants #1 and #3, assessments were conducted at their bedside. Several interruptions (from medical team or roommate) and room configuration prevented the collection of a complete dataset.

#### Sensory evaluation

**Fig. 1:**
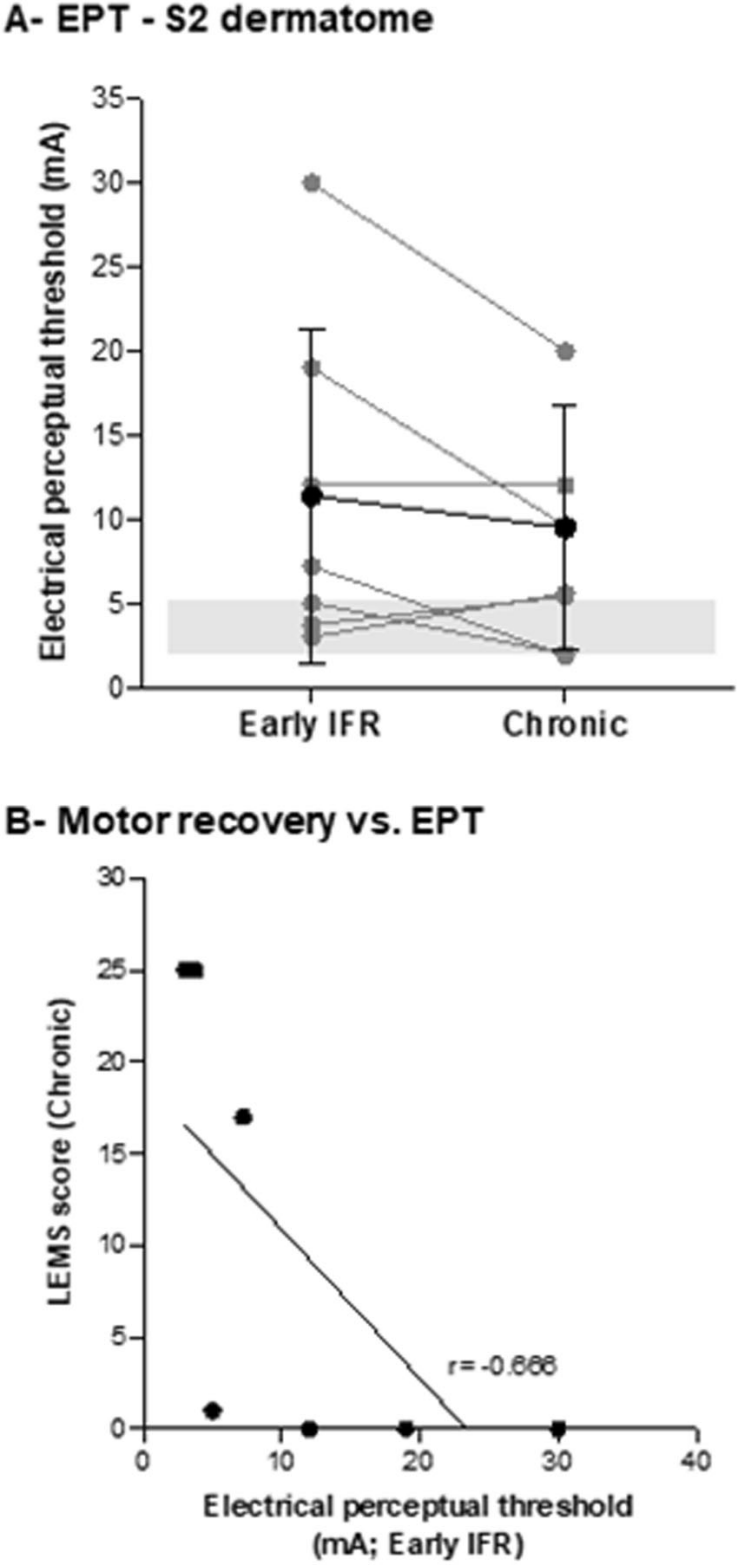
Assessment of the electrical perceptual threshold. **A** Electrical perceptual thresholds (EPT) of right and left S2 dermatomes at the early IFR and chronic assessment time points. Grey points correspond to SCI participants. The lines indicate values from the same participant at both time points. Black points correspond to the mean of SCI participants ± SD. The grey shaded rectangle shows data from control participants. **B** Correlation between motor recovery (LEMS at the chronic stage) and EPT assessed at early IFR.

The values of the average EPTs of both limbs at the S2 dermatome of the SCI were higher in SCI participants both in the early (Cohen’s *d* = 1.37) and chronic phases (Cohen’s *d* = 1.33) compared to controls. However, no differences were observed in the average EPT of SCI participants between the early IFR and chronic phases (Cohen’s *d* = 0.22). Figure 1A shows the evolution of EPT values, as a function of time, for both legs. Data for the control participants are represented as a grey box. Supplementary Table 1 details the values for each SCI participant. Participant #5 (AIS A) could not detect the stimulus at any of the intensities used and is thus not represented in these graphs. LEMS scores in the chronic stage were obtained for all participants and Pearson correlation analysis showed *r* = −0.666 between early IFR EPT scores and LEMS scores in the chronic stage (Fig. 1B).

#### H-Reflex

The H/M ratio in SCI participants, either at early IFR or at the chronic stage (right = 0.4 ± 0.3, left = 0.5 ± 0.4), were similar to control participants (right leg = 0.4 ± 0.2; left leg = 0.3 ± 0.2; Cohen’s *d* early IFR vs. control = 0.28; Cohen’s *d* chronic vs. control = 0.29). The H_max_ latency in SCI participants were similar at early IFR and chronic, but occurred later than controls (Cohen’s *d* early IFR vs. control = 0.83; Cohen’s *d* chronic vs. control = 0.48)

**Fig. 2:**
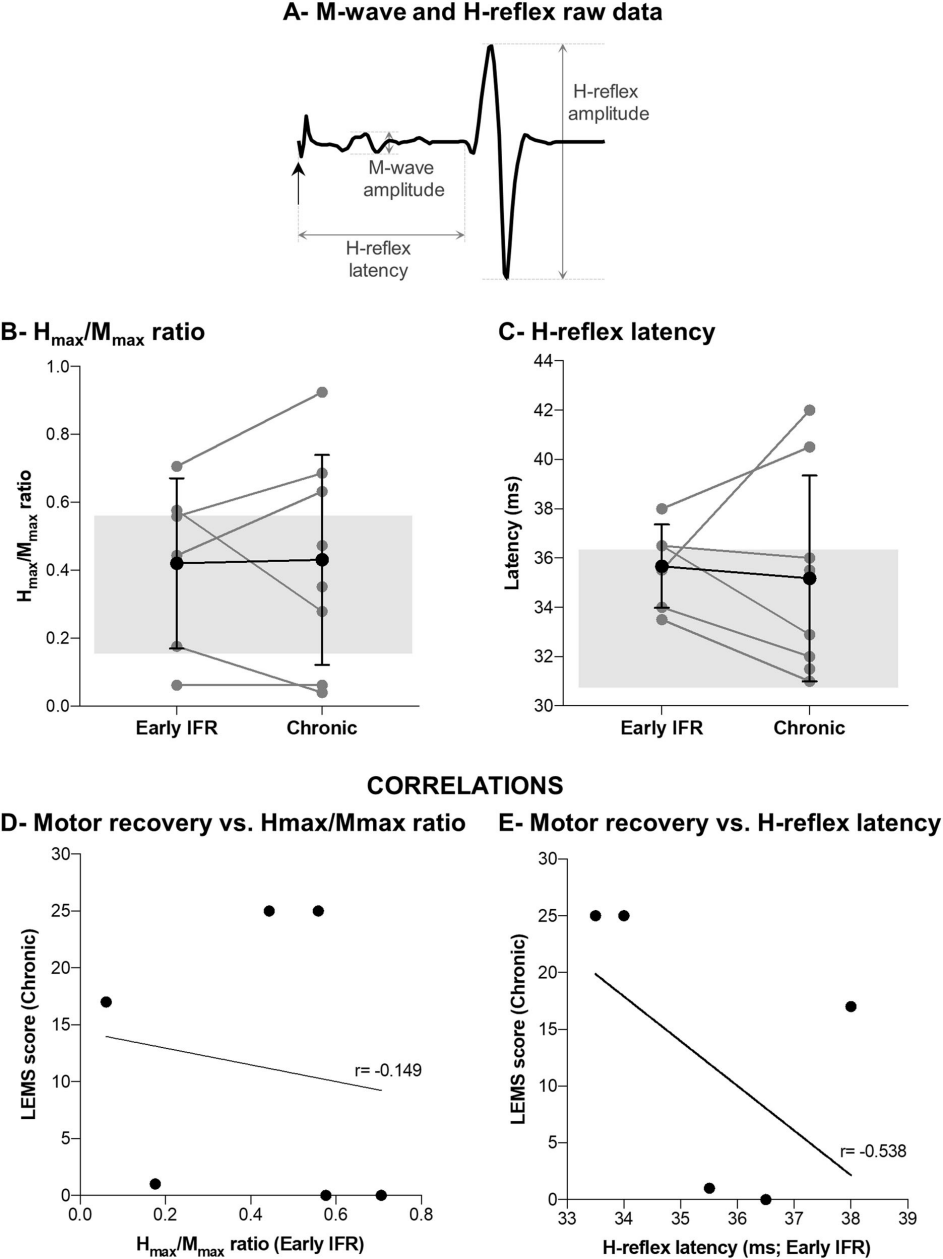
Assessment of the Soleus H-Reflex. **A** Typical M-wave and H-wave on a control participant’s SOL EMG induced by tibial nerve stimulation. **B**, **C** The light grey dots and lines represent individual SCI participants, and the black dots and error bars represent the mean of SCI participants. The shaded grey rectangle encompasses the mean ± SD of control participants. **D**, **E** Lack of correlation between motor recovery and H/M ratio (D) or H_max_ latency (E) assessed at early IFR.

Figure 2A represents the measures acquired, and Fig. 2B, C show the evolution of the H/M ratio and H-reflex latency as a function of time for each SCI participant. Neither the ratio nor latency at early IFR correlates with the LEMS score in the chronic phase (*r* = −0.149 and −0.538 respectively; Fig. 2D, E). Supplementary Table 2 compiles the H-reflex characteristics of each SCI participant.

#### Motor evoked potential

**Fig. 3:**
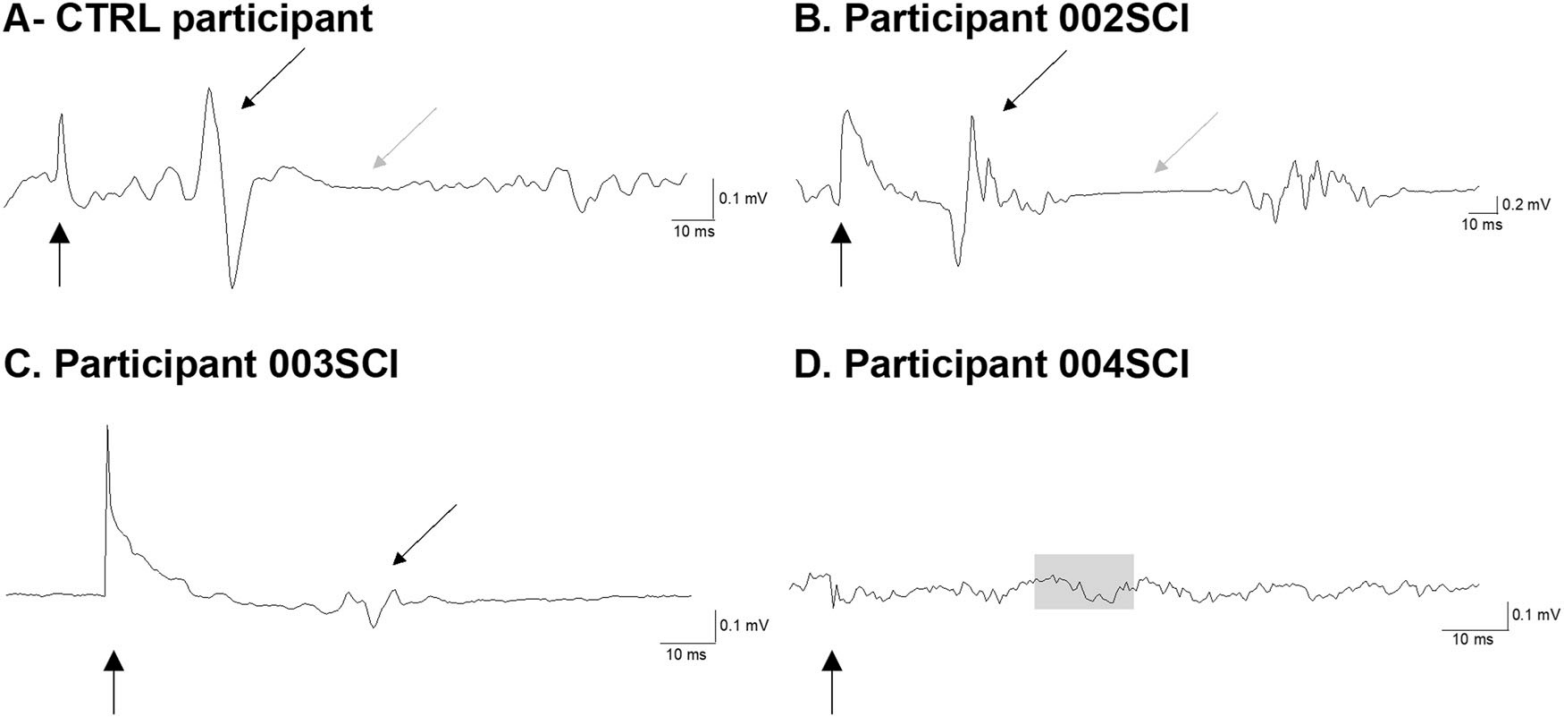
MEP assessment in Tibialis Anterior. Motor evoked potential in a control (**A**) and three SCI participants (**B**, **C**, **D**) recorded in the tibialis anterior muscle. The black dotted arrows point to the MEP and the grey dotted arrows point to the silent period. The dashed grey box in (**D**) indicates where the MEP was expected as no MEP was observed in this participant. The solid black arrows indicate when the stimulation was applied.

Figure 3 illustrates a MEP in one control and three SCI participants. MEPs could be elicited at all time points in SCI participant #2 (AIS D). In SCI participant #3 (AIS B), a clear MEP was only visible in the left leg during the chronic phase, although the participant was able to slightly move their leg at early IFR. No MEP was elicited in SCI participant #4 (AIS B) at either time point. MEP latencies seem to be longer and MEP amplitudes smaller in SCI participants compared to controls, and only SCI participant #2 had a measurable silent period (SP). Supplementary Table 3 details the MEP characteristics of these participants.

#### MRI

**Fig. 4:**
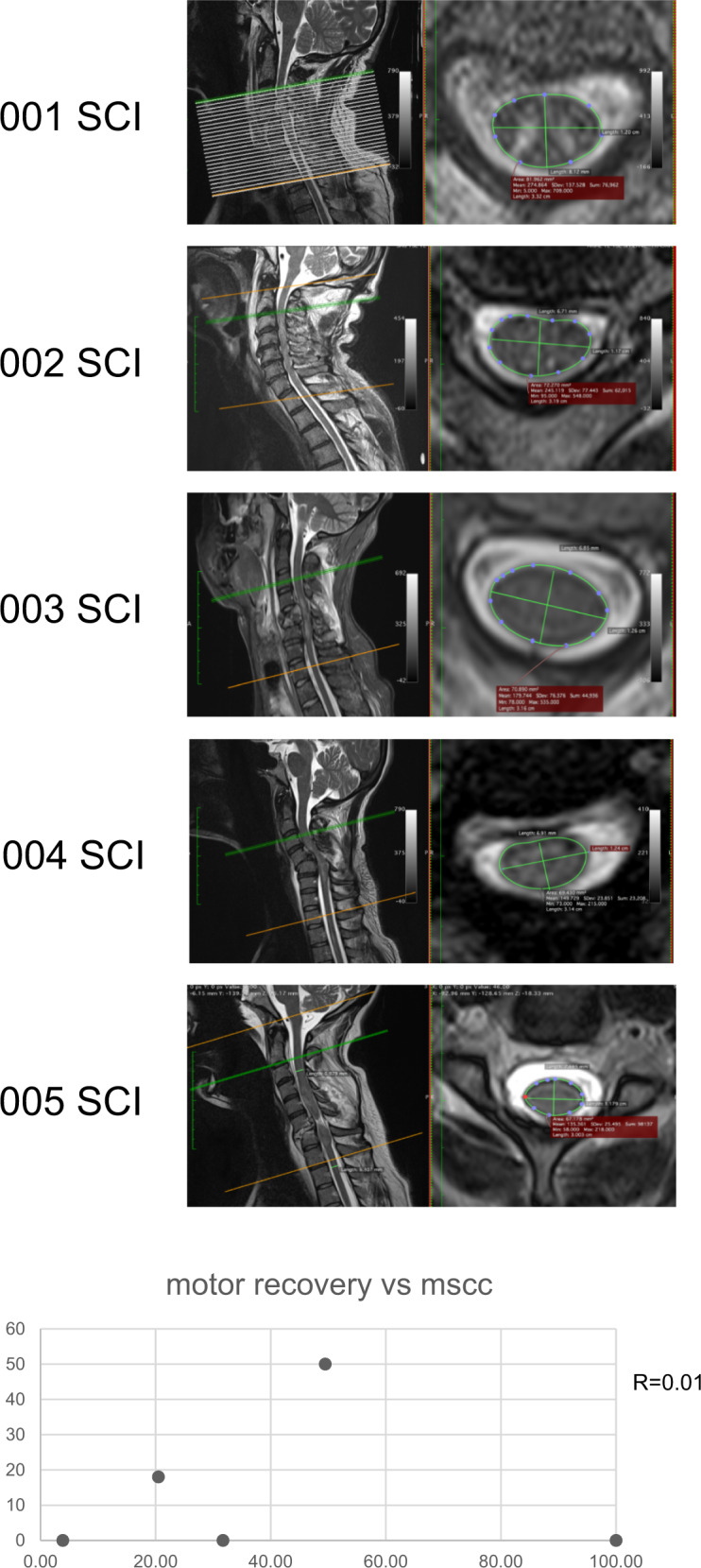
Magnetic resonance imaging of the spinal cord. Upper panel: this figure illustrates the spinal cord images of the five participants of the pilot study as well as the localisation of the measures. Lower panel: Correlation between motor recovery (LEMS at the chronic stage) and MSCC assessed at early IFR.

Figure 4A illustrates the MRI for each participant as well as where the measures were taken. In Fig. 4B, no clear trend was observed between the MSCC taken at admission and bilateral LEMS. Supplementary Table 4 shows the MSCC values for each participant.

## Discussion

Overall, the preliminary results suggest that an early multimodal electrophysiological assessment protocol is feasible and support the relevance of exploring the use of electrophysiological methods in a future large-scale study.

### Feasibility

Our results show that these techniques can be safely applied in early IFR. Furthermore, the data collected on the feasibility criteria prompted modifications of the experimental protocol, notably splitting the assessment into two sessions to enable collection of a complete dataset.

### Could statistical models be developed to predict recovery of SCI patients based on electrophysiological means?

The neurophysiological techniques in the current paper have been tested before and were shown to be valid to measure spinal cord excitability in specific pathways. However, these measures are not used clinically in a hospital setting. Recent studies trying to determine algorithms to predict motor recovery have been based on more clinical tools (such as INSCSCI) in their model. However, more precise measures, such as the ones used in this project, could better reflect actual neurological damage and might be relevant to include in a statistical model, but this has never been tested so far.

Hence, the novelty of this pilot study is to propose an assessment protocol combining multimodal electrophysiological and MRI measures that could be readily implemented within an IFR setting and enable the identification of neural pathways that were spared by the lesion. Together with clinical means, this information might lead to the development of a robust predictive model of recovery.

### Impact of combined electrophysiological and MRI measures

#### Electrical perceptual threshold

The EPT of the S2 dermatome was able to discriminate between sensory capacities of SCI participants even between participants with similar AIS category and level (participants 3 and 4). It was also predictive of motor recovery of the lower limb. However, care should be exercised when considering this conclusion, given the small number of participants tested. Our results echo those of previous studies that have shown that EPTs in individuals with SCI were significantly higher than control participants [24, 25, 28–31].

#### H-Reflex

Neither the H_max_/M_max_ ratio nor the H_max_ latency were discriminant between SCI participants and may not be predictive for functional outcomes of the lower limb on their own. The absence of the H-reflex in early IFR was not predictive of an absent H-reflex in subsequent periods. However, participants with an absent H-reflex had either very high or absent EPT, reflecting a stronger impairment of spinal excitability This conclusion supports previous findings showing that H-reflex excitability differences depend on the severity of the lesion (completeness of injury) [32–37].

#### Motor evoked potential

In this pilot study, a MEP could be elicited in two of the three participants tested once they could voluntarily activate their muscle. However, the MEP could not be elicited when muscle activity was weak, in one participant. Although this result echoes that of previous studies [38], others have demonstrated the predictive potential of MEP for motor recovery [39–46]. In the current pilot study, the coil might have been a limiting factor, as a simple figure of 8 coil was used instead of a conical coil [20].

Nonetheless, when MEPs were elicited, the amplitude and latency reflected the severity of corticospinal damage.

#### Magnetic resonance imaging

MSCC analysis did not reflect the motor potential of patients in the long-term. However, the patient whose MSCC value was 100, reflecting no quantifiable connections, was also the only participant without any EPT at S2 and SOL H-reflex. This suggests that the combination of electrophysiology and MR techniques could lead to the identification of patients with the most severe lesions and smaller potential of recovery. However, this preliminary finding would need to be replicated in a larger study to be confirmed.

#### Neuronal correlates of these findings

By specifically documenting the neuronal pathways spared at least in part by the lesion, probable deficits and potential for recovery might be better assessed. Studies in animal models have demonstrated that a lesion in the dorsolateral part of the spinal cord interrupts the corticospinal tract and results in paw drag [47], while a ventral lesion interrupts vestibulospinal and reticulospinal tracts and results in balance and weight support deficits [48, 49]. These pioneering animal studies link the location of the lesion, the interrupted pathways, and the functional deficits. They also hint at the potential of plasticity within the remaining pathways, since all the animals were able to recover quadrupedal locomotion. Moreover, recent studies showed extensive somatosensory and motor corticospinal sprouting following an incomplete SCI [50–52]. The recovery potential will depend on the ability of remaining neuronal circuitry to generate new and coordinated activity through neuroplasticity and could lead to compensation.

### Limits of this pilot study

Only the LEMS was used in this pilot project. However, future studies should include both upper and LEMSs (total motor scores) to develop a more global portrait of abilities in individuals with SCI.

Furthermore, in this pilot study we focused on electrophysiological measurements as we wanted to verify whether it was realistic to undertake these measures in such a subacute environment. However, behavioural measurements should be examined with the neurophysiological measurements for more complete assessment.

Only a few electrophysiological measures were used in the current study. Corticospinal pathways and sensory pathways were assessed as they may be interrupted to different degrees by the lesion and induce different levels of impairment. We postulated that knowing the degree to which the pathway is impaired soon after the injury could give an idea of the actual damage that occurred as well as the potential for recovery. The H-reflex is a more indirect measure where we assessed excitability below the level of lesion. Indeed, previous studies have suggested changes in neuronal excitability below the level of the lesion [32–37] and we wanted to assess whether there was a correlation between excitability in neuronal networks below the lesion and motor recovery. However, it would be essential to collect other data, such as those related to pain and spasticity, for predictive purposes, which will be targeted in the longitudinal study.

This pilot study showed that a multimodal electrophysiological assessment protocol is feasible in a rehabilitation setting after a traumatic SCI. The importance of electrophysiological methods to supplement clinical examinations is not new and has been previously described [14, 53, 54]. Statistical models could be based on a combination of neurophysiological measures to determine with greater precision the completeness of the lesion and the potential for recovery. In the next phase, a larger-scale, longitudinal study will generate patterns of electrophysiological response for individuals with SCI, as well as their predictive value.

## Supplementary information

Supplementary data

## Data Availability

All data supporting the findings of this study are available within the article and its Supplementary Materials.
