# Supplementary material for: Quantitative electrophysiological assessments as predictive markers of lower limb motor recovery after spinal cord injury: a pilot study with an adaptive trial design
Source: Spinal Cord Ser Cases. 2022 Feb 24;8:26. doi: 10.1038/s41394-022-00491-0 (PMC8873458; doi:10.1038/s41394-022-00491-0)
Supplement: Supplementary file 1 — Supplementary data [file 41394_2022_491_MOESM1_ESM.pdf]

Supplementary Table 1. Compilation of participant's mean EPT values at the S2 dermatome over time for the right leg (A) and left leg (B).

A)

**Right S2 dermatome**		
	Early IFR	Chronic
001		20
002	3	5.6
003	5	2
004	12	12
005	absent	absent
Control average	3.6 ± 1.2	

B)

**Left S2 dermatome**		
	Early IFR	Chronic
001	30	20
002	3.75	5.4
003	7.2	1.9
004	19	9.6
005	absent	absent
Control average	3.5 ± 2.0	

Note: An absent EPT means that the participant did not feel anything in their lower limbs at stimulation intensities > 40 mA. Shaded areas indicate that EPT was not tested.

IFR, intensive functional rehabilitation; EPT, electrical perceptual threshold; S, sacral level

Supplementary Table 2. Characteristics of participants' H-reflex over time.

H/M ratio

**Right H/M ratio**		
	Early IFR	Chronic
001SCI		0.472
002SCI	0.559	0.686
003SCI	0.176	0.040
004SCI	0.576	0.279
005SCI	absent	absent
Control average	0.4 ± 0.2	

**Left H/M ratio**		
	Early IFR	Chronic
001SCI	absent	0.351
002SCI	0.443	0.632
003SCI	0.061	0.062
004SCI	0.706	0.924
005SCI	absent	absent
Control average	0.3 ± 0.2	

H_{max} latency

**Right H_{max} latency**		
	Early IFR	Chronic
001SCI		35.5
002SCI	34.0	32.0
003SCI	35.5	42.0
004SCI	36.5	32.9
005SCI	absent	absent
Control average	33.3 ± 3.0	

**Left H_{max} latency**		
	Early IFR	Chronic
001SCI	absent	31.5
002SCI	33.5	31.0
003SCI	38.0	40.5
004SCI	36.5	36.0
005SCI	absent	absent
Control average	34.0 ± 2.7	

IFR, intensive functional rehabilitation; **H_{max}**,,maximal H wave; shaded areas = H-reflex was not tested;

Supplementary Table 3. Characteristics of participants' MEPs across time

		Early IFR		Chronic	
**Participants**	**Variables**	Right leg	Left leg	Right leg	Left leg
**002**	MEP (yes/no)	yes	yes	yes	yes
	Stim. Intensity (%)	100	100	90	90
	MEP-A (mV)	0.292 ± 0.135	0.392 ± 0.066	0.915 ± 0.231	0.88 ± 0.203
	MEP-A (%Mmax)	NA	NA	30.1 ± 7.59	40.73 ± 9.4
	MEP-L (ms)	47.7 ± 7.6	32.6 ± 1.25	40.42 ± 5.29	35.03 ± 2.85
	SP (yes/no)	yes	yes	yes	yes
	SP-Latency (ms)	71,7	74	58,2	52,5
	SP-Duration (ms)	150,9	208	72,51	54,48
	Area ratio (%)	50.3	95.1	49.2	63
**003**	MEP (yes/no)	no	no	yes	yes
	Stim. Intensity (%)			94	94
	MEP-A (mV)			0.063 ± 0.03	0.073 ± 0.03
	MEP-A (%Mmax)			NA	NA
	MEP-L (ms)			40.29 ± 2.45	50.501 ± 5.11
	SP (yes/no)			no	no
	SP-Latency (ms)				
	SP-Duration (ms)				
	Area ratio (%)				
**004**	MEP (yes/no)	no	no	no	no
	Stim. Intensity (%)				
	MEP-A (mV)				
	MEP-A (%Mmax)				
	MEP-L (ms)				

	MEP during contraction		MEP at rest	
**Control average**	MEP (yes/no)	yes	MEP (yes/no)	yes
	Stim. Intensity (%)	48 ± 4	Stim. Intensity (%)	63 ± 5
	MEP-A (mV)	0.36 ± 0.10	MEP-A (mV)	0.24 ± 0.14
	MEP-A (%Mmax)	14. 7 ± 3.0	MEP-A (%Mmax)	7.8 ± 2.6
	MEP-L (ms)	33.4 ± 0.9	MEP-L (ms)	34.6 ± 1.3
	SP (yes/no)	yes	SP (yes/no)	No clear SP
	SP-Latency (ms)	53.7 ± 2.4	SP-Latency (ms)	
	SP-Duration (ms)	62.8 ± 14.9	SP-Duration (ms)	
	Area ratio (%)	55.1 ± 7.9	Area ratio (%)	

MEP: motor evoked potential; MEP-A: Amplitude of MEP; MEP-L: Latency of MEP
SP: silent period; IFR, intensive functional rehabilitation
NA: the Mmax was not assessed, so the normalized MEP was not evaluated

Supplementary Table 4. MSCC value and combined LEMS

	MSCC	LEMS (R+L)
001SCI	3,89	0
002SCI	49,46	50
003SCI	20,50	18
004SCI	31,70	0
005SCI	100,00	0
